# COVID-19 and Gastrointestinal Tract: From Pathophysiology to Clinical Manifestations

**DOI:** 10.3390/medicina59101709

**Published:** 2023-09-24

**Authors:** Filippo Vernia, Hassan Ashktorab, Nicola Cesaro, Sabrina Monaco, Susanna Faenza, Emanuele Sgamma, Angelo Viscido, Giovanni Latella

**Affiliations:** 1 Gastroenterology Unit, Division of Gastroenterology, Hepatology, and Nutrition, Department of Life, Health and Environmental Sciences, University of L’Aquila, Piazzale Salvatore Tommasi 1, 67100 L’Aquila, Italy; filippo.vernia1@gmail.com (F.V.); dott.nicolacesaro@gmail.com (N.C.); monaco.sa6@gmail.com (S.M.); susannafaenza@yahoo.it (S.F.); emanuelesgamma94@gmail.com (E.S.); angelo.viscido@univaq.it (A.V.); 2 Department of Medicine, Gastroenterology Division, Howard University College of Medicine, Washington, DC 20060, USA; hashktorab@howard.edu

**Keywords:** COVID-19, SARS-CoV-2, gastrointestinal tract, pathophysiology of COVID-19

## Abstract

*Background*: Since its first report in Wuhan, China, in December 2019, COVID-19 has become a pandemic, affecting millions of people worldwide. Although the virus primarily affects the respiratory tract, gastrointestinal symptoms are also common. The aim of this narrative review is to provide an overview of the pathophysiology and clinical manifestations of gastrointestinal COVID-19. *Methods*: We conducted a systematic electronic search of English literature up to January 2023 using Medline, Scopus, and the Cochrane Library, focusing on papers that analyzed the role of SARS-CoV-2 in the gastrointestinal tract. *Results*: Our review highlights that SARS-CoV-2 directly infects the gastrointestinal tract and can cause symptoms such as diarrhea, nausea/vomiting, abdominal pain, anorexia, loss of taste, and increased liver enzymes. These symptoms result from mucosal barrier damage, inflammation, and changes in the microbiota composition. The exact mechanism of how the virus overcomes the acid gastric environment and leads to the intestinal damage is still being studied. *Conclusions*: Although vaccination has increased the prevalence of less severe symptoms, the long-term interaction with SARS-CoV-2 remains a concern. Understanding the interplay between SARS-CoV-2 and the gastrointestinal tract is essential for future management of the virus.

## 1. Introduction

Since the first report in December 2019 in Wuhan, China, coronavirus disease 2019 (COVID-19) spread all over the world, causing more than 758 million cases and 6.85 million deaths [1].

The clinical course of Severe Acute Respiratory Syndrome Coronavirus 2 (SARS-CoV-2) infection ranges from asymptomatic to a rapidly progressing and life-threatening disease and is associated with a variety of symptoms [2].

Like other coronaviruses, SARS-CoV-2 infects the gastrointestinal tract, inducing nausea, vomiting, abdominal pain, and diarrhea [3,4,5].

Since the COVID-19 occurrence in late 2019, intense research efforts on an unprecedented scale have focused on the study of SARS-CoV-2 entry mechanisms and clinical presentations.

Prior to COVID-19, there were two short-lived pandemics—SARS-CoV-1 in 2002 and MERS in 2012. The first cases of SARS-CoV-1 were detected in China and quickly spread with a high lethality rate of 11%, resulting in 8422 reported cases and 916 deaths. Similarly, MERS emerged in Saudi Arabia with a high mortality rate of approximately 37% and was also traced back to bats. Both viruses caused similar symptoms such as fever, cough, dyspnea, and atypical pneumonia, as well as affecting the gastrointestinal tract with diarrhea being a common symptom. However, MERS had a higher prevalence of GI symptoms, mortality rate, and need for extreme treatment measures such as mechanical ventilation compared to SARS-CoV-1. Despite the potential for fecal–oral transmission, extrapulmonary symptoms were not given much attention due to the short-lived and localized nature of these pandemics [6,7].

The aim of this narrative review is to compile a selection of relevant papers to summarize the current evidence on the pathophysiology of gastrointestinal SARS-CoV-2 infection, fecal–oral route transmission, clinical manifestations, and the outcomes of patients with gastrointestinal symptoms.

## 2. Materials and Methods

A systematic electronic search of the English literature up to January 2023 was performed using Medline, EMBASE, Scopus, and the Cochrane Library. The search strategy used a combination of Medical Subject Headings (MeSH) and keywords as follows: COVID-19; SARS-CoV-2; gastrointestinal symptoms; intestinal symptoms; gastrointestinal infection; intestinal infection; intestinal replication; long COVID; post-acute COVID syndrome.

Five authors (F.V., N.C., S.M., S.F., E.S.) identified relevant articles by screening the abstracts. Additional studies were selected after a manual review of the reference list of the identified studies and review articles. Any discrepancy was resolved by consensus, referring to the original articles. Out of 4783 citations, 147 relevant articles were selected and included in the present narrative review.

## 3. Prevalence of Gastrointestinal Symptoms

Several studies reported gastrointestinal symptoms in patients affected by COVID-19. Their prevalence in adults is high, with diarrhea, nausea, and abdominal pain being the most frequent ones (16.5%, 9.7%, 4.5%, respectively) [8]. Anorexia or loss of appetite (1.6%), vomiting (1.5%), and loss of taste (1.3%) are less common. Increased liver enzymes are not rare (5.6%) (Table 1) [8]. Prevalences, however, are hardly comparable as different series do not report all main gastrointestinal symptoms. This likely results from the different weight attributed by authors to mild and/or infrequent symptoms, which were not invariably reported.

In the pediatric population, the incidence of gastrointestinal symptoms is higher compared to adults [9]. A recent meta-analysis reported a higher prevalence of nausea/vomiting (19.7%) and abdominal pain (20.3%) but not diarrhea (19.08%) [9]. However, wide variations have been described in different series [10].

The relationship between gastrointestinal symptoms, mortality, and more severe systemic disease presentation is debatable. Available evidence suggests that the overall presence of gastrointestinal symptoms is not associated with increased mortality rates (OR = 0.88; 95% CI 0.71–1.09; *p* = 0.23). The same applies to individual symptoms, such as diarrhea (*p* = 0.96), nausea/vomiting (*p* = 0.46), and abdominal pain (*p* = 0.3) [11].

A recent meta-analysis correlating the incidence of gastrointestinal symptoms with severe presentation in adults showed that abdominal pain (OR = 2.70, 95% CI 1.17–6.27, *p* = 0.02), but not diarrhea, nausea, or vomiting, is associated with aggressive disease [12].

In the pediatric population, the presence of diarrhea significantly correlates with more severe clinical course (OR 3.9, 95% CI: 1.80–8.73; *p* < 0.01). This is not the case for nausea/vomiting and abdominal pain [9].

The persistence of gastrointestinal symptoms for more than two weeks after discharge is also high, as 3.23% of patients reported nausea, 3.19% vomiting, and 4.12% prolonged diarrhea, while the persistence of abdominal pain was 1.68% [13].

Gastrointestinal bleeding (GIB) was reported in 2% of COVID-19 patients, respectively, 1% for upper GI and 1% for lower GI [14]. The most common causes were ulcers (25.3%), erosive/ulcerative diffuse damage (16.1%), and petechial/hemorrhagic gastropathy (9.2%) in upper gastrointestinal bleeding. Ischemic colitis was reported in one-third of patients with lower gastrointestinal bleeding [15]. Again, some differences have been reported in different cohorts [4]. The presentation with GIB however represents a negative prognostic factor, as reported by a recent systematic review comparing the outcomes of 808 COVID-19 patients with GIB and 18,179 non-GIB COVID-19 patients. The overall incidence of GIB was 0.06%, with GIB patients showing a higher death rate than non-GIB patients (25.4% vs. 16.4%, *p* < 0.001) [4].

## 4. Gastrointestinal Infection

Respiratory droplets are the main route of transmission of SARS-CoV-2 [16], but SARS-CoV-2 RNA was also detected in fecal samples [17,18,19,20].

Direct evidence of fecal–oral transmission is still lacking, but emerging evidence supports the hypothesis [21,22]. SARS-CoV-2 RNA has indeed been detected in anal swabs and stool samples in over 50% of infected patients [23,24,25]. RNA levels in the stools ranged from 102 to 105 copies/mL, but in several reports fecal shedding exceeded 107 copies/mL [23,26,27,28]. This is in line with nasopharyngeal fluids concentration (105–1011 copies/mL) [27,28]. SARS-CoV-2 concentration in stool samples peaks 2 to 3 weeks after symptom onset [25,29], and, as reported in a small German cohort, the RNA load in fecal specimens reflects what is found in sputum in 86% of cases (6 of 7 patients) [23]. Live SARS-CoV-2 was also observed in the feces of COVID-19 patients, confirming potential fecal–oral transmission [30]. However, despite being easily detected by electron microscopy [31], SARS-CoV-2 isolation from stools is difficult [32].

Recent evidence suggests that viral variants show different gastrointestinal infectivity. Reduced viral replication of Omicron BA.1 and BA.2 variants compared with the B.1.617.2/Delta variant was reported in studies carried out in organoids [33].

The persistence of the virus in stools is significantly longer (median 22 days, interquartile range 17–31 days) than in respiratory (18 days, 13–29 days; *p* = 0.02) and serum samples (16 days, 11–21 days; *p* < 0.001) [25]. Polymerase chain reaction detected viral RNA in fecal samples of patients with no detectable virus in respiratory tract specimens [19,25,34].

Several studies have shown the presence of SARS-CoV-2 in epithelial cells of both the small and large intestines, as demonstrated by intestinal biopsies [17,35,36]. Additionally, the transcription of subgenomic SARS-CoV-2 mRNA (sgmRNA) indicates active viral replication in the intestine, as sgmRNA is only transcribed in infected cells and not packaged into virions [23]. Intestinal organoids primarily secrete SARS-CoV-2 apically [37], which may explain viral excretion in feces.

Although evidence suggests that the gut is an active site of SARS-CoV-2 replication, it is still unclear whether the virus present in feces is directly infectious. While viral RNA may be shed in fecal specimens, the presence of viral ribonucleic acid does not necessarily imply the presence of live transmissible virus [38]. Therefore, while direct and indirect data support the hypothesis that SARS-CoV-2 actively infects human intestinal epithelial cells, further research is needed to determine whether the virus in feces is directly infectious.

## 5. SARS-CoV-2 Structure and Interaction with the Host

**Figure 1:**
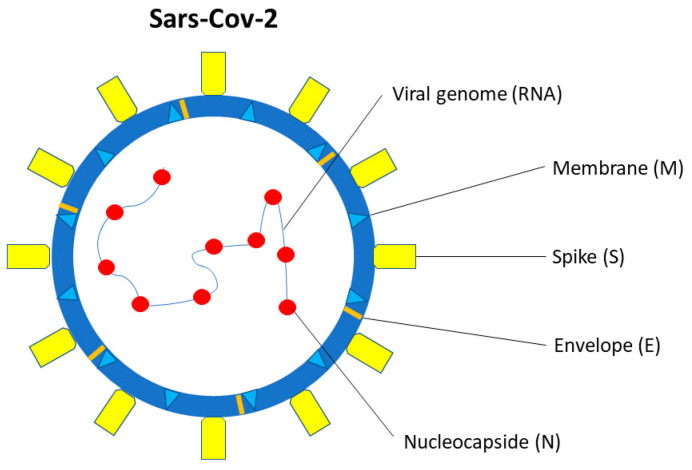
SARS-CoV-2 structure. SARS-CoV-2 is a single-stranded β-coronavirus. Two-thirds of viral RNA, in the first open reading frame (ORF 1a/b), encodes for 16 non-structural proteins (NSP). The remaining viral genome expresses accessory proteins, interfering with the host innate immune response, and four structural proteins (S, E, N, M).

SARS-CoV-2 is a single-stranded β-coronavirus, with 29.9 kb RNA genome and an envelope with spikes on the surface (Figure 1) [39]. It shares up to 80% of the gene sequence with other pathogenic members of the coronavirus family, such as SARS-CoV-1 and Middle East Respiratory Syndrome coronavirus (MERS-CoV) [40].

Two-thirds of viral RNA, in the first open reading frame (ORF 1a/b), translates two polyproteins, encoding for 16 non-structural proteins (NSP). The remaining viral genome expresses accessory proteins, interfering with the host innate immune response, and structural proteins. The four essential structural proteins include the spike (S) glycoprotein, small envelope (E) protein, nucleocapsid (N) protein, and matrix (M) protein [41]. Proteins M, E, and S form the viral envelope [42]. The N protein is structurally bound to the viral RNA and is involved in viral replication [43], while the M protein plays a role in determining the shape of the viral envelope [43].

Open reading frames encoding nine accessory proteins (3a, 3b, 6, 7a, 7b, 8, 9b, 9c, and 10) and two polyproteins (pp1a and pp1ab) are also present in the genome [44].

Polyproteins pp1a and pp1ab, but not accessory proteins, are involved in viral replication [44].

**Figure 2:**
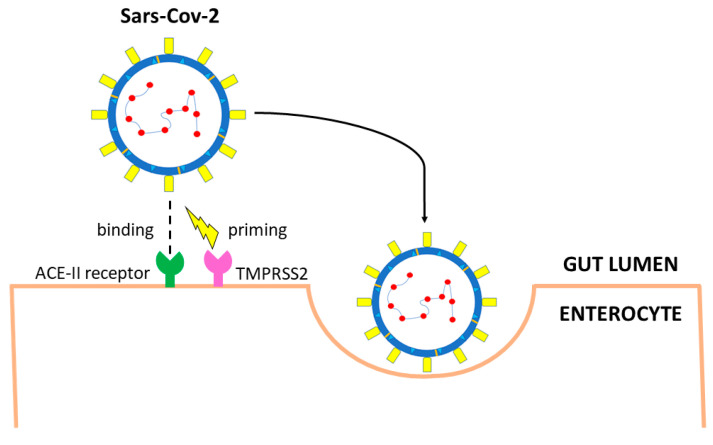
Main entry route of SARS-CoV-2 in gastrointestinal tract. The spike protein of SARS-CoV-2 binds angiotensin-converting enzyme 2 (ACE2) receptors. Priming by TMPRSS2, TMPRSS4 (or other proteases such as FURIN) leads to endocytosis. The virus is uncoated, genomic RNA is released, and viral proteins are synthesized using the host replication system.

SARS-CoV-2 interacts with target cells through envelope spike glycoprotein which binds the angiotensin-converting enzyme 2 (ACE2) receptor of the host (Figure 2) [41]. The binding affinity for the human ACE2 receptor is 10–20 times stronger for SARS-CoV-2 than for its predecessor SARS-CoV-1 [45]. After binding ACE2 receptors, the transmembrane serine protease 2 (TMPRSS2) mediates the cleavage of the spike glycoprotein, regulating the internalization of the virus into target cells [46]. The two subunits, S1 and S2, favor the binding of the virus to the cell and the fusion between the two cellular membranes [46].

Two proteolytic events are however needed to activate SARS-CoV-2. The first is the cut in the specific cleavage site between the S1 and S2 domains, which is recognized by various proteases, including TMPRSS2 [46] and furin [47,48]. The second cleavage site is within the S2 domain and allows the exposition of the fusion peptide, which enables membrane fusion. This second cleavage can be either performed by TMPRSS2 on the surface of the host cell or by lysosomal proteases, such as cathepsin L in the endolysosomes [49]. Following the entry into the cell, the virus is uncoated and replicates using the host replication system [42].

## 6. Route of Gastrointestinal Infection

The exact route of infection is still undefined, but the virus likely reaches the gut after being swallowed.

In vitro studies show that SARS-CoV-2, like other enveloped viruses, loses infectivity after 10 min incubation in gastric fluid [50]. Nonetheless, this route of transmission is possible as other viruses, such as influenza virus, despite being vulnerable to digestive juices, retain infectivity when protected by highly viscous mucus [51].

The glycosylation of the S protein is a further mechanism by which other coronaviruses survive the adverse milieu of the stomach and bile-salt-containing duodenal juice [52]. The mechanism may be shared by SARS-CoV-2.

Indirect evidence deriving from in vitro models of other coronaviruses suggests that fasting or fed state modulate infectivity. Indeed, MERS rapidly loses infectivity in simulated fasting state, low pH gastric fluid, but not after 2 h of exposure to fed-state condition [53].

It has also been hypothesized that chronic H. pylori infection leading to atrophic gastritis and intestinal metaplasia could facilitate SARS-CoV-2 intestinal infection by reducing stomach acidity [54,55]. H. pylori also increases the expression of ACE-2 receptors in the GI tract [56]. Clinically, a strong correlation between the occurrence of abdominal pain (19.4% vs. 2.6%, *p* = 0.007) or diarrhea (32.3% vs. 9.1%, *p* = 0.006) and H. pylori infection has been reported in COVID-19 patients [57].

Similarly, proton pump inhibitor-induced hypochloridria could favor SARS-CoV-2 intestinal infection [58], but available data are conflicting. Patients on PPI had significantly higher requirement for oxygen therapy, intensive care unit admission, and invasive ventilation than patients not taking PPI (fully adjusted OR (aOR): 2.39; 95% CI: 1.08–5.10) in a post hoc analysis from a nationwide Korean cohort [5]. Conversely, several meta-analyses did not confirm these early reports [59,60].

## 7. Gastrointestinal Tract–Virus Interaction

Lungs are the primary route of SARS-CoV-2 infection, but ACE2 receptors, an 805 amino acids-, type I cell-surface glycoprotein [61], are highly expressed also on the brush border of the enterocytes [62,63]. The ACE2 receptor is detected by immunofluorescent staining also in the glandular cells of the stomach and colon [17]. Viral RNA has been detected also in the esophageal mucosa, but the lack of viral nucleocapsid protein staining suggests a low viral load [17]. This is in keeping with low ACE2 expression in squamous esophageal epithelial cells. ACE2 receptors are minimally expressed by enteroendocrine cells, Paneth cells, and goblet cells [61,64,65,66].

Under physiological conditions, ACE2 receptors in the gastrointestinal tract are associated with the amino acid carrier B0AT1, which regulates the homeostasis of tryptophan, and thus stimulates the production of mechanistic target of rapamycin (mTOR)-dependent antimicrobial peptides from Paneth cells [67,68].

Besides ACE2 receptors, other molecules may play a role in SARS-CoV-2 infection. It has been demonstrated that TMPRSS2 is highly expressed in the gastrointestinal tract [50], not only in enterocytes [50] but also in intestinal goblet [69] and Paneth cells (Figure 2) [70,71]. Other serine proteases of the same family, such as TMPRSS4, are also highly expressed in mature enterocytes and potentially favor SARS-CoV-2 infection [50]. An additive effect of the two enzymes has been documented, suggesting synergic effect resulting from distinct cellular and subcellular localization of the two proteases [50].

The protease furin, widely present in the stomach, small bowel, and colon [72], also enables the S protein to separate into two pinching structures [73]. Although furin significantly increases the cleavage of the S protein, promoting SARS-CoV-2 infectivity and spread, its presence is not essential [74].

In addition to proteases, other alternative entry molecules, such as neuropilin-1 (NRP1), have been identified [75]. The expression of NRP1 in the small bowel has been reported on both human biopsies and organoids [76,77]. Following protease cleavage of the S protein into S1 and S2, a polybasic Arg-Arg-Ala-Arg carboxyl-terminal sequence on S1 binds NRP1 [75,78]. The role of NRP1 is less clear than ACE2 receptors, but the protein might mediate SARS-CoV-2 infection in ACE2-negative cells [77] and possibly explain different disease behavior in adults and children [76].

Recent studies suggest a possible interaction between the SARS-CoV-2 S protein and the cluster of differentiation (CD) 147 binding site [79], possibly through cyclophilin A-mediated regulation of ACE2 receptors [80], but conflicting results have also been published [81].

A small Italian case series [82] suggested that VEGF through CD147 may trigger gastrointestinal ischemia in SARS-CoV-2, as reported in other conditions [83]. However, validation in larger series is needed.

Protease-mediated membrane fusion represents the usual SARS-CoV-2 entry route in the host cell. An alternative option has recently been advocated, consisting of endocytosis resulting from SARS-CoV-2 binding to ACE2 receptors followed by S protein cleavage by cathepsin L [49] expressed in a variety of tissues including the gastrointestinal tract [84,85].

## 8. Gastrointestinal Damage, Inflammation, and Symptoms

**Figure 3:**
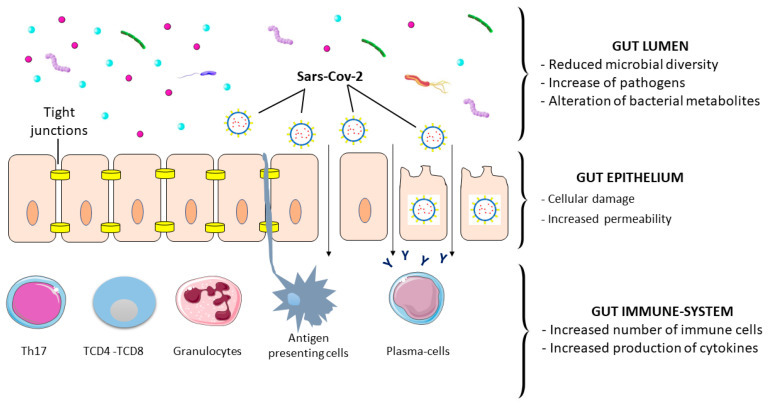
SARS-CoV-2–gastrointestinal tract interactions. SARS-CoV-2 infection induces a disruption of intestinal barrier integrity. Gut microbiota is altered with a reduction in microbial diversity, reduction in beneficial bacterial strains, and an increase in pathogens. The mechanical barrier, consisting of intestinal epithelial cells and tight junctions, is also affected by the infection. SARS-CoV-2 induces immune activation, resulting in increased levels of cytokines and leukocytes.

SARS-CoV-2 is responsible for direct and indirect damage of the gastrointestinal system and symptoms (Figure 3).

SARS-CoV-2 induces syncytia formation in human enteroids [50]. Independently of other viral components, the S protein induces cell fusion in vitro [50]. Syncytia formation is cytopathic, resulting in loss of integrity of the intestinal barrier, and favors cell-to-cell viral spread and immune-response evasion [50]. Epithelial damage is also related to reduced expression of tight junction marker genes, such as ZO-3 and CLDN1 [86].

In vivo intragastric inoculation with SARS-CoV-2 reduces cell proliferation and increases apoptosis of intestinal epithelial and goblet cells in an animal model of SARS-CoV-2 infection [87]. Other in vitro studies on Caco-2 cells confirmed viral replication but did not report cytopathic effects [88]. It should also be considered that data from in vitro models using tumoral cell lines with different protein expression need confirmation in human biopsies.

Duodenal biopsies collected during upper endoscopy in a small case series of COVID-19 patients with GI symptoms were characterized by villous blunting and increased intraepithelial lymphocytes [89]. This supports the view that intestinal damage is mediated by the immune response, more than by direct viral damage, and may explain the lack of cytopathic effect reported in some models.

The enhanced migration of immune cells to the intestinal mucosa during SARS-CoV-2 infection is in line with a study using mass cytometry [36]. Reduced dendritic cells in the lamina propria were also reported [36] but not in studies using mass cytometry in postmortem tissue samples [90].

Infection likely triggers innate immune response in the intestine through the recognition of pathogen-associated molecular patterns (PAMPs), as reported in the lung, leading to recruitment of immune cells and favoring adaptative response [91,92].

SARS-CoV-2 infection increases the expression of proinflammatory genes such as IL-1b, IL-6, CCL2, CCL3, CCL5, and CXCL10 in human small intestinal epithelial cells derived from pluripotent stem cells [86]. The S1 spike protein stimulates IL-6 and IL-8 secretion in CACO-2 human intestinal epithelial cells [93]. Some studies also reported an increased release of intestinal IL-18 [94,95]. Interestingly, elevated IL-18 levels correlate with disease severity [95,96].

In vitro, intestinal epithelial cells were reported to produce type III interferon (IFN) in response to SARS-CoV-2 infection, leading to less effective viral replication [97]. Other studies on IFN-III had opposite findings [37].

SARS-CoV-2 infection induces the inflammatory response in the gut [98], in turn increasing the likelihood of intestinal symptoms. COVID-19 patients with diarrhea have higher concentrations of fecal calprotectin (FC) compared to patients without diarrhea (123.2 ± 58.8 vs. 17.3 ± 4.8, *p* < 0.001) [98]. Interestingly, FC levels significantly correlate with serum IL-6 (*p* < 0.001) but not with C-reactive protein [98].

The pathogenic role of SARS-CoV-2 on enterocytes is indirectly supported by plasma citrulline concentrations [99]. The molecule is almost exclusively produced by enterocytes and is not incorporated into proteins, thus representing a biomarker of small bowel enterocyte mass and function [100]. The accepted citrulline cutoff level for the diagnosis of the short bowel syndrome (<20 μmol/L) has been found in 61.5% of patients from a small cohort, and 15.4% had concentrations below 10 μmol/L [99], suggesting severe intestinal damage. Correlation of low citrulline levels with digestive symptoms (62.5% vs. 20%, *p* = 0.05) and PCR (84.5 vs. 13.5 mg/L, *p* = 0.03) was also present [99].

High concentrations of serotonin (5-HT) were documented in COVID-19 patients with diarrhea compared to those who did not present with the symptom, or healthy controls [101]. The role of 5-HT in regulating GI motility and inflammation is well known [102], but its involvement in SARS-CoV-2-associated diarrhea is undocumented.

Intestinal inflammation is associated with severe systemic disease as fecal calprotectin levels were increased in patients with abnormal chest X-ray findings [103].

Active intestinal inflammation in vivo in COVID-19 patients is supported by high fecal levels of the pro-inflammatory IL-8 and low fecal levels of anti-inflammatory IL-10 compared to controls [104]. Fecal IL-23 levels were high in patients with severe COVID-19 disease [104].

SARS-CoV-2-specific IgA antibodies were high in patients with severe disease [104]. Their clinical importance is unclear, as a longitudinal study showed that IgA and IgM antibodies rapidly decay, but not IgG antibodies, which are detectable up to 105 days after symptom onset [105].

The role of adaptative immune response in GI symptoms has been reported by several studies. COVID-19 patients with diarrhea have increased CD3+ and higher CD4+ T cell counts compared to patients without diarrhea. As CD8+ T cell counts were lower in the diarrhea group, the CD4/CD8 ratio was increased. High counts of CD19+ B cells and low CD16+CD56+ natural killer (NK) cells were also reported in diarrhea [106].

## 9. SARS-CoV-2 and Gut Microbiota Alteration

**Figure 4:**
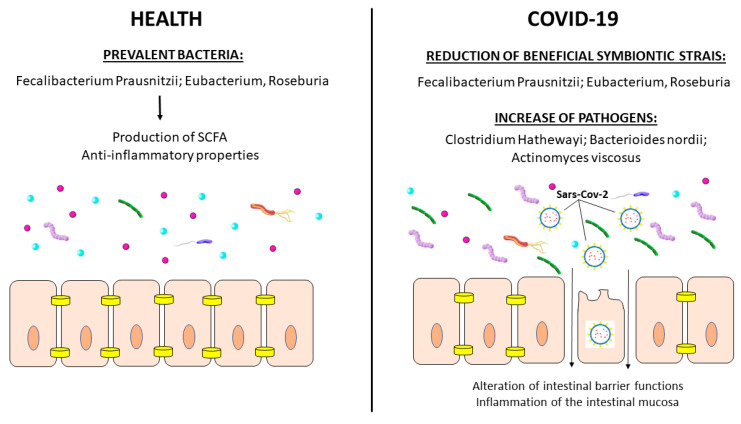
Gut microbiome alterations in COVID-19. In healthy individuals, Faecalibacterium prausnitzii, Eubacterium and Roseburia are the prevalent strains of gut microbiome. These bacteria show anti-inflammatory properties and produce SCFA. In patients affected by COVID-19, a depletion of symbionts and an increase in opportunistic pathogens were described. Gut dysbiosis observed during COVID-19 infection persists after SARS-CoV-2 clearance.

Gut microbiota changes have been linked to gastrointestinal symptoms. Several studies investigated gut microbiota through 16S rRNA gene-amplified sequencing in COVID-19 patients [107]. Antibiotic-naïve patients with COVID-19 showed higher concentrations of opportunistic pathogens, including Actinomyces viscosus, Clostridium hathewayi, and Bacteroides nordii, compared to controls (Figure 4). A recent meta-analysis of 16 studies confirmed a significant reduction in alpha diversity in COVID-19 patients compared to controls (standardized mean difference, SMD = −0.78; 95% CI, −1.25 to −0.31) [107]. Changes persisted after recovery (SMD = −1.14; 95% CI, −1.60 to −0.68) [107]. Depletion of beneficial symbiotic bacterial strains was observed in patients on antibiotics [108].

Interestingly, a small study comparing microbiota dysregulation in patients affected by H1N1 influenza A and COVID-19 identified specific microbial signatures in the two diseases, with H1N1 displaying lower diversity and different overall microbial composition [109].

Gut microbiota alterations influence the metabolism of several compounds and negatively affect infection rates and symptoms. Reduced gut microbiome metabolism of tryptophan was reported in patients with COVID-19 and GI symptoms [110]. Tryptophan is absorbed by the intestinal epithelial cell B0AT1/ACE2 transporter and indirectly regulates the expression of antimicrobial peptides [111]. It may be anticipated that SARS-CoV-2-binding intestinal ACE2 receptors might reduce tryptophan absorption and ultimately modify the microbiota [112].

Analysis of the bacterial function in 66 antibiotics-naïve COVID-19 patients and 70 controls showed that patients with severe disease are characterized by significant (*p* < 0.001) impairment of short-chain fatty acid (SCFA) and L-isoleucine biosynthesis that persists over 30 days after recovery [113]. The reduction in SCFA and L-isoleucine significantly correlated with disease severity and levels of inflammation-related proteins such as CXCL-10, NT-proB-type natriuretic peptide, and C-reactive protein (*p* < 0.05). This reflects a functional modification of intestinal microbiome. However, in vitro studies aimed at the evaluation of the anti-inflammatory effects of butyrate led to contrasting results [114,115]. In vivo butyrate supplementation studies to improve symptoms have not been carried out.

Some of the role of microbiota on SARS-CoV-2 outcomes is indirectly supported by the finding that the genus Collinsella, producing ursodeoxycholate and other secondary bile acids, was negatively correlated with mortality in a Japanese cohort [116]. The underlying rationale resides in the reduced farnesoid X receptor signaling with consequent downregulation of ACE2 receptor transcription, which decreases susceptibility to SARS-CoV-2 in vitro, in vivo, and in human lungs and livers perfused ex situ [117].

## 10. Chronic GI Tract Diseases and SARS-CoV-2

The mechanisms by which SARS-CoV-2 affects the gastrointestinal tract of healthy subjects are still largely unclear, more so considering that the host–virus bidirectional relation may modify both the course of infection and the preexisting disease.

ACE2 and TMPRSS2 are overexpressed in inflammatory bowel disease (IBD) patients [118,119,120]. However, cytoplasmic and membrane ACE2 are significantly higher in ulcerative colitis (UC) compared to healthy controls. In Crohn’s disease (CD), the difference is observed only in the membrane [118]. Moreover, colonic ACE2 expression is lower in CD than in UC (*p* < 0.0001) [121].

Increased expression of ACE2 and TMPRSS2 in inflamed versus non-inflamed colonic and ileal segments has been reported by some authors [122] but not by others [118], casting doubt on the modulation of receptor expression induced by active disease.

Moreover, circulating components of the alternative renin–angiotensin system, ACE2 and angiotensin (1–7), were increased in a small series of patients with IBD [123], possibly playing a protective role against SARS-CoV-2 infection [124].

Potentially clinically relevant differences have been reported for other proteins involved in the infection. TMPRSS2 is overexpressed in IBD [118,122,125], while furin levels are significantly lower in colonic specimens of UC patients versus controls [126]. The clinical relevance of these findings is at best debatable as clinical databases, including SECURE-IBD [127], Dutch [128], and Danish [129] series, did not report increased COVID-19 severity in IBD patients. Furthermore, the incidence rate of COVID-19 is not increased in IBD patients undergoing immunomodulant therapy [130].

Differences in relevant epithelial protein expression are present in other chronic diseases. ACE2 is overexpressed in the stomach of patients with chronic gastritis [54,55,131] and underexpressed in eosinophilic esophagitis and gastroenteritis [132]. However, to our knowledge, no association with outcome has been reported [133,134,135].

Overall, poor general conditions, resulting from concomitant disease, are by far more relevant in worsening clinical course and outcome than the expression of receptors or the existence of immune-related illnesses [136].

## 11. Gastrointestinal Symptoms and Long COVID

COVID-19 is associated with long-term gastrointestinal symptoms, persisting in up to 8.4% of patients at 3 months and in 6.6% at 6 months [137]. Schmulson proposed new criteria for defining post-COVID-19 functional gastrointestinal disorders (FGID) as follows. Patients should not meet the criteria for FGID before COVID-19, symptoms should fulfill Rome IV criteria and persist after the resolution of acute SARS-CoV-2 infection [138].

The alterations underlying the development of post-infective FGID are still undefined but likely reside in persistent subclinical inflammation, increased intestinal permeability, and microbiota changes. It is known that irritable bowel syndrome (IBS) may develop after an acute infection (e.g., Campylobacter, Shigella, Salmonella, Giardia, Noroviruses) [139,140,141], and the same might apply to SARS-CoV-2.

A 6-month prospective study in post-acute COVID-19 syndrome reported high levels of Ruminococcus gnavus and Bacteroides vulgatus and low levels of Faecalibacterium prausnitzii. Butyrate-producing bacteria showed significant inverse correlations with occurrence of the syndrome [142], but it is unclear whether modifications of the intestinal flora precede or are caused by long COVID.

As far as gut permeability is concerned, the concentration markers, such as the fatty acid binding protein 2, were higher in COVID-19 patients compared to controls (*p* = 0.0013) [143]. Zonulins and cadherins are also likely involved [144,145].

Incomplete viral clearance or persistence of viral antigens is an additional mechanism triggering symptoms, and it appears that the S1 protein alone is sufficient to stimulate the production of inflammatory cytokines in vitro [93]. Patients with persistent viral shedding in the stool were more likely to report nausea (aOR = 1.61, CI 95% = 1.09–2.39), vomiting (3.20, 1.11–9.21), and abdominal pain (2.05, 1.09–3.86) but not diarrhea (1.10, 0.63–1.91) [146]. Moreover, the presence of SARS-CoV-2 antigens in the colon was documented 257 days following diagnosis, in the absence of ongoing fecal shedding [147].

Patients who have received the bivalent vaccine for COVID-19, which combines two different vaccine types, may experience different outcomes compared to those who have received the monovalent vaccine, which contains only one type of vaccine. While both vaccines have been shown to be effective in preventing COVID-19 infection, post-bivalent vaccine patients may have a higher level of protection against certain strains of the virus [148]. Additionally, post-bivalent vaccine patients may experience a different side-effect profile than monovalent vaccine patients due to the different types of vaccines used in the combination. However, further research is needed to fully understand the differences between these two vaccine types and their impact on patients’ health. Ultimately, regardless of the type of vaccine received, vaccination remains a critical tool in the fight against the COVID-19 pandemic. In addition to the potential differences in vaccine effectiveness and side-effect profiles, there may also be differences in the gastroenterology symptoms experienced by patients who have received bivalent versus monovalent vaccines. Some studies have suggested that COVID-19 infection can lead to gastrointestinal symptoms such as nausea, vomiting, and diarrhea. While the vaccines themselves do not typically cause these symptoms, it is possible that post-bivalent vaccine patients may experience a different gastrointestinal symptom profile due to the different types of vaccines used in the combination. However, more research is needed to fully understand the potential differences in gastrointestinal symptoms between these two vaccine types. Regardless, it is to know that long-hauler symptoms are less in people infected with Omicron who have received the bivalent vaccine [149,150,151].

## 12. Conclusions

SARS-CoV-2 infection dramatically changed the worldwide health scenario. Following the initial impact characterized by acute severe infection and high mortality rate, the virus is now becoming endemic due to massive vaccination campaigns and disease-induced antibodies. We will thus be facing a long-term interaction with the SARS-CoV-2 virus, with increased prevalence of less aggressive symptoms.

Epidemiological and clinical data indicate that in the first phase of epidemics, gastrointestinal symptoms were frequent, occurring in up to one-third of patients. The figure will increase as the virus becomes endemic.

The rapid global spread of this new, highly aggressive virus prompted fast sharing of biomedical knowledge. This often resulted in redundant information and the publication of review articles sometimes reporting unconfirmed or questionable data.

The present narrative review provides an updated overview of SARS-CoV-2 gastrointestinal infection and symptoms and the pathophysiological mechanism of intestinal damage. This may prove of some use for physicians treating SARS-CoV-2 patients in clinical practice. However, most hard information derives from preclinical/in vitro studies, whereas clinical and epidemiological studies are mainly retrospective, underpowered, lack reliable protocols, and the length of follow-up is necessarily short.

Thus, major questions regarding the interaction of SARS-CoV-2 and the GI tract are still unanswered. It is unclear how the virus survives in the gastric environment, which cell types are primarily infected in vivo, and to which extent preexisting intestinal disease modifies susceptibility, clinical course, and prognosis. Similarly, the long-term effects of post-acute COVID-19 syndrome are still to be determined, but it is likely that, as after other infections, post-infective functional disorders may develop. The results of well-designed ongoing studies are needed to answer these questions and provide reliable insight into the GI effects of SARS-CoV2 infection. The bivalent COVID-19 vaccine, combining two different vaccine types, offers a higher level of protection against new virus strains compared to the monovalent vaccine. It is presently undefined whether the two vaccine types or Omicron-derived new virus strains are associated with changing prevalence or severity of side effects or differences in the profile of gastrointestinal symptoms.

In conclusion, gastrointestinal symptoms associated with SARS-CoV-2 infection are usually mild and self-limiting. Symptomatic treatment may be needed in case of nausea and vomiting. Rehydration associated to mild diet may prove useful in some patients with diarrhea, while loperamide and other anti-diarrheal drugs are likely best avoided as in other gastrointestinal infections.

Clinical trials using dietary modification, probiotics, prebiotics, and fecal microbiota transplantation are underway to determine their effectiveness in the treatment of acute COVID-19 and possibly enhance the effectiveness of SARS-CoV-2 vaccines [152,153,154,155,156,157,158]. Only long-term clinical data and future research shall provide better insight into the complex bidirectional interaction between the virus and the alimentary tract in healthy patients and those with preexisting gastrointestinal diseases.

## Figures and Tables

**Table 1:** Gastrointestinal symptoms in adult and pediatric patients.

	Adult Patients	Pediatric Patients
Diarrhea	16.5%	19.0%
Nausea/vomiting	11.2%	19.7%
abdominal pain	4.5%	20.3%
Anorexia	1.6%	10%

Data reported in two recent meta-analyses by Shehab [8] and Bolia [9] for adults and children, respectively.

## Data Availability

Not applicable.
